# Fasting for 24 Hours Heightens Reward from Food and Food-Related Cues

**DOI:** 10.1371/journal.pone.0085970

**Published:** 2014-01-16

**Authors:** Jameason D. Cameron, Gary S. Goldfield, Graham Finlayson, John E. Blundell, Éric Doucet

**Affiliations:** 1 School of Human Kinetics, University of Ottawa, Ottawa, Ontario, Canada; 2 Children's Hospital of Eastern Research Institute Ottawa, Ottawa, Ontario, Canada; 3 University of Leeds, Leeds, United Kingdom; Monash University, Australia

## Abstract

**Introduction:**

We examined the impact of a 24 hour complete fast (vs. fed state) on two measures of food reward: 1) ‘wanting’, as measured by response to food images and by the relative-reinforcing value of food (RRV), and 2) ‘liking’, as measured by response to food images and the hedonic evaluation of foods consumed.

**Methods:**

Utilizing a randomized crossover design, 15 subjects (9 male; 6 female) aged 28.6±4.5 yrs with body mass index 25.3±1.4 kg/m^2^ were randomized and counterbalanced to normal feeding (FED) and 24-hour fast (FASTED) conditions. Trait characteristics were measured with the Three Factor Eating Questionnaire. Two computer tasks measured food reward: 1) RRV progressive ratio task, 2) explicit ‘liking’ and ‘wanting’ (Leeds Food Preference Questionnaire, LFPQ). Also measured were *ad libitum* energy intake (EI; buffet) and food ‘liking’ (visual analogue scale) of personalized stimuli.

**Results:**

There were no significant anthropometric changes between conditions. Appetite scores, hedonic ratings of ‘liking’, and *ad libitum* EI all significantly increased under the FASTED condition (p<0.05). Under the FASTED condition there were significant increases in the RRV of snack foods; similarly, explicit ‘wanting’ and ‘liking’ significantly increased for all food categories. ‘Liking’ of sweet foods remained high across-meals under FASTED, but savory foods decreased in hedonic saliency.

**Conclusion:**

Relative to a fed state, we observed an increase in hedonic ratings of food, the rewarding value of food, and food intake after a 24 hr fast. Alliesthesia to food and food cues is suggested by heightened hedonic ratings under the FASTED condition relative to FED.

## Introduction

In one of the best known and most controlled studies of human feeding it was shown that upon re-feeding after 24 weeks of energy restriction (approximately 1500 kcal/day), subjects who volunteered for the Minnesota Semi-starvation Study demonstrated chronic hyperphagia, obsessive preoccupations with sweet foods and food images, and a host of negative reactions dubbed “semistarvation neurosis” [1]. Similar findings have been reported regarding aberrant feeding with acute periods (1 day) of fasting in clinical populations [2], [3]. Others, however, have found that acute fasting does not necessarily result in excessive energy intake or negative affect upon reinitiating *ad libitum* feeding in non-clinical subjects [4], [5]. It is still unclear what factors underpin compensatory eating and altered hedonics in the early stages energy deprivation. There are conflicting data, but it appears that the short-term manipulation of satiety, most often achieved with a pre-load paradigm, does not reliably impact palatability [6]–[8]. While the preload paradigm may be sensitive to possible short-term signaling of need (free)-state, a better manipulation has been less well studied and involves increasing the deprivation state through sustained energy deprivation. In support of the external validity of a sustained energy deprivation, it can be pointed out that recent NHANES data show that 63% of respondents were trying to lose weight [9], and older data has shown that approximately 14% of Americans have reported using short-term fasting as a means of losing weight [10]. Furthermore, the relevance of studying subjects under periods of deprivation longer than a few hours is reflected in the observation that day-to-day feeding typically occurs in response to learned cues [11], and not necessarily to a need-state.Thus, although it is clear that under challenges of negative energy balance feeding behavior can be altered with or without measurable changes in body energy reserves, what remains to be better described are the psychological and behavioral factors that drive changes in appetite, feeding, and food reward under such conditions.

Feeding behavior has been shown to be modulated by hunger-state, whereby prolonging inter-meal intervals or inducing weight loss can positively influence perceived taste pleasantness, a concept called alliesthesia [12], [13]. Food reward can be operationalized as stimuli (internal and external) that contribute to the pleasure of and motivation to obtain food. Indeed, rigorous studies involving animal and human models of feeding have led to considerable progress in the understanding of the psychology of food reward [14] and the neural substrates that mediate it (for review see [15], [16]). In order to further examine the potential clinical importance of food reward in body weight regulation, and to better describe the overlapping pathways of food and drug reward [17], there has been a surge in studies examining hunger states (*e.g.* high hunger vs. sated) with brain imaging [18]–[23]. Concomitantly, there has been an emergence of standardized objective tools to measure psychological constructs of food reward (*e.g.* hedonics of food and food-related stimuli [24], [25] and food reinforcement [26]). Energy deprivation not only impacts the palatability—or ‘liking’—of a particular food stimulus [27]–[29], but also impacts the desire—or ‘wanting’—to engage a food stimulus [30]. In utilizing a computer task that applies behavioral economic theory to human feeding [31], ongoing research on the reinforcing value of food has demonstrated that ‘wanting’ can change in a state-dependent manner (*i.e.* hungry vs. satiated) [32]–[34]. Similarly, several groups have demonstrated in human subjects that the hedonic ‘liking’ evaluation of ingested foods can be impacted acutely by hunger-state [35], [36] or chronically by a state of reduced body energy reserves [37], [38]. Taken together, the dual qualities of food reward—‘wanting’ and ‘liking’—can be described as susceptible to changes in the internal milieu, analogous to Michel Cabanac's concept of alliesthesia [13]. It is unclear, however, what impact an acute 24 hour fasting challenge will have on the direction of ‘wanting’ and ‘liking’ for preferred food items or whether a relationship exists with changes in appetite, food reward and *ad libitum* EI.

There is a growing interest in fasting and alternate day (modified) fasting as methods to achieve weight loss or simply as a way of living (*e.g.* religious fasting periods). The main objective of this randomized crossover study was to examine the impact of a 24 hour complete fast (vs. fed state) on appetite, food reward, and EI. It was hypothesized that under the fasting condition there would be significantly higher appetite scores and *ad libitum* EI, and that correlations would exist between these changes. It was further hypothesized that relative to the fed state, snack food would become more rewarding, with increased explicit ‘wanting’ and ‘liking’, and the meal-induced attenuation of explicit ‘liking’ for the fasted condition would be less pronounced.

## Methods

### Subjects

Fifteen volunteers (9 male; 6 female) aged 28.6±4.5 yrs. with body weight 74.7±4.9 kg and body mass index (BMI) 25.3±1.4 kg/m^2^ (*i.e.* normal weight/overweight) participated in this randomized crossover study, once in the fed state (FED) and once after performing a 24-hour complete fast (FASTED). Subjects were free from any illnesses and medication that could have influenced the outcome of the experiment and met the following inclusion criteria: non-diabetic, non-smokers, not pregnant, weight stable for ≥6 months (±2 kg) and aged between 18 and 40 years. Only pre-menopausal women with a regular menstrual cycle (28–35 days) were recruited including those using oral contraceptives. Characteristics of subjects under control (FED) and experimental (FASTED) conditions are presented in **Table 1**. This study was conducted according to the guidelines laid down in the Declaration of Helsinki and the University of Ottawa Research Ethics Committee approved all procedures involving human subjects. Written informed consent was obtained from all subjects.

**Table 1:** Subjects' characteristics under FED (control) and FASTED (experimental) conditions.

Characteristic	FED	FASTED	%Change	Time	Sex	Time x Sex
**Anthropometric**						
Body Weight (kg)	74.4±4.9	74.2±4.9	0.3	0.12	0.10	0.60
BMI (kg/m^2^)	25.2±1.4	25.0±1.4	0.8	0.09	0.56	0.69
**Appetite (AUC)**						
Desire to Eat	555.1±316.9	719.2±123.3	30.5	0.05	0.32	0.79
Hunger	415.6±169.5	703.5±121.8	69.3	0.001	0.36	0.08
Fullness	560.5±44.4	295.6±100.1	47.3	0.001	0.32	0.12
PFC	492.4±43.5	741.6±131.5	50.6	0.001	0.14	0.75
**Food Hedonics**						
Snack	120.5±7.6	135.1±4.9	12.1	0.005	0.70	0.94
Fruit	112.3±7.1	130.9±5.0	16.6	0.02	0.25	0.44
Pizza 1	107.9±29.9	119.6±23.9	10.8	0.05	0.90	0.98
Pizza 2	95.6±37.2	118.6±28.9	24.1	0.005	0.90	0.20
Dessert	121.1±8.6	132.2±4.9	9.2	0.06	0.67	0.91
AUC	436.6±79.4	502.6±68.8	15.1	0.001	0.58	0.26
**RRV**						
Snack Points	16.9±9.3	25.6±12.4	51.5	0.008	0.90	0.26
Snack Responses	381.9±202.2	613.5±344.3	60.6	0.03	0.99	0.14
Fruit Points	33.1±9.4	23.7±11.9	28.4	0.03	0.98	0.37
Fruit Responses	201.6±52.1	145.1±77.3	28.0	0.008	0.95	0.09
***Ad Libitum*** ** EI**						
Total EI (grams)	300.0±42.4	388.8±55.2	29.6	0.001	0.021	0.30
Total EI (kcal)	491.1±99.6	854.9±104.6	74.1	0.001	0.003	0.35
Energy Density	1.65±0.2	2.4±0.2	45.5	0.002	0.55	0.76
%Sugar	17.6±1.9	29.0±7.5	64.8	0.06	0.19	0.71
%Fat	6.5±1.1	12.2±3.7	87.7	0.21	0.42	0.35
%Protein	2.4±0.35	4.7±9.0	95.8	0.05	0.76	0.26

Appetite scores were measured hourly and pre-and post food consumption with 150 mm visual analogue scales (VAS). Similarly, the change in food hedonics was measured by VAS ratings for palatability immediately following the ingestion of preferred foods. Food reinforcement was measured with a behavioral choice computer task.

Area under the curve (AUC) was calculated with the trapezoid method and included all variables described in the Food Hedonics category. Note that BMI is body mass index; PFC is prospective food consumption; EI is energy intake in kilocalories (kcal); Energy Density is kcal/gram of food consumed; and RRV is relative-reinforcing value. Values are means ± SD.

### Design and procedure

#### Screening visit

The study began during the initial (screening) visit to the laboratory, where written informed consent was obtained and then height and body weight were measured. Each subject was then asked to complete a questionnaire to indicate his or her favorite snack food and favorite fruit/or vegetable. For this decision, they were asked to circle five choices each from a comprehensive list of common food products (chips, candies, cakes, and chocolate bars) and common fruits/vegetables, or if the choice was not on the list, they were asked to indicate what their favorite food items were. In this manner each subject would have his single favorite snack food and fruit/or vegetable as reinforcers for the RRV paradigm. Note that no subjects chose a vegetable as their preferred food item, so all non-snack foods were fruits. Also, subjects were required to complete the Three Factor Eating Questionnaire (TFEQ). In order to minimize the hormonal effects on main outcomes, measurements for women were scheduled between days 1–5 of the menstrual cycle, where ovarian hormones are at their lowest levels [39]. As such, women had at least a 1-month period, and men at least a 2-week period between the FED and FASTED sessions.

### Experimental manipulation of FED state

**Figure 1:**
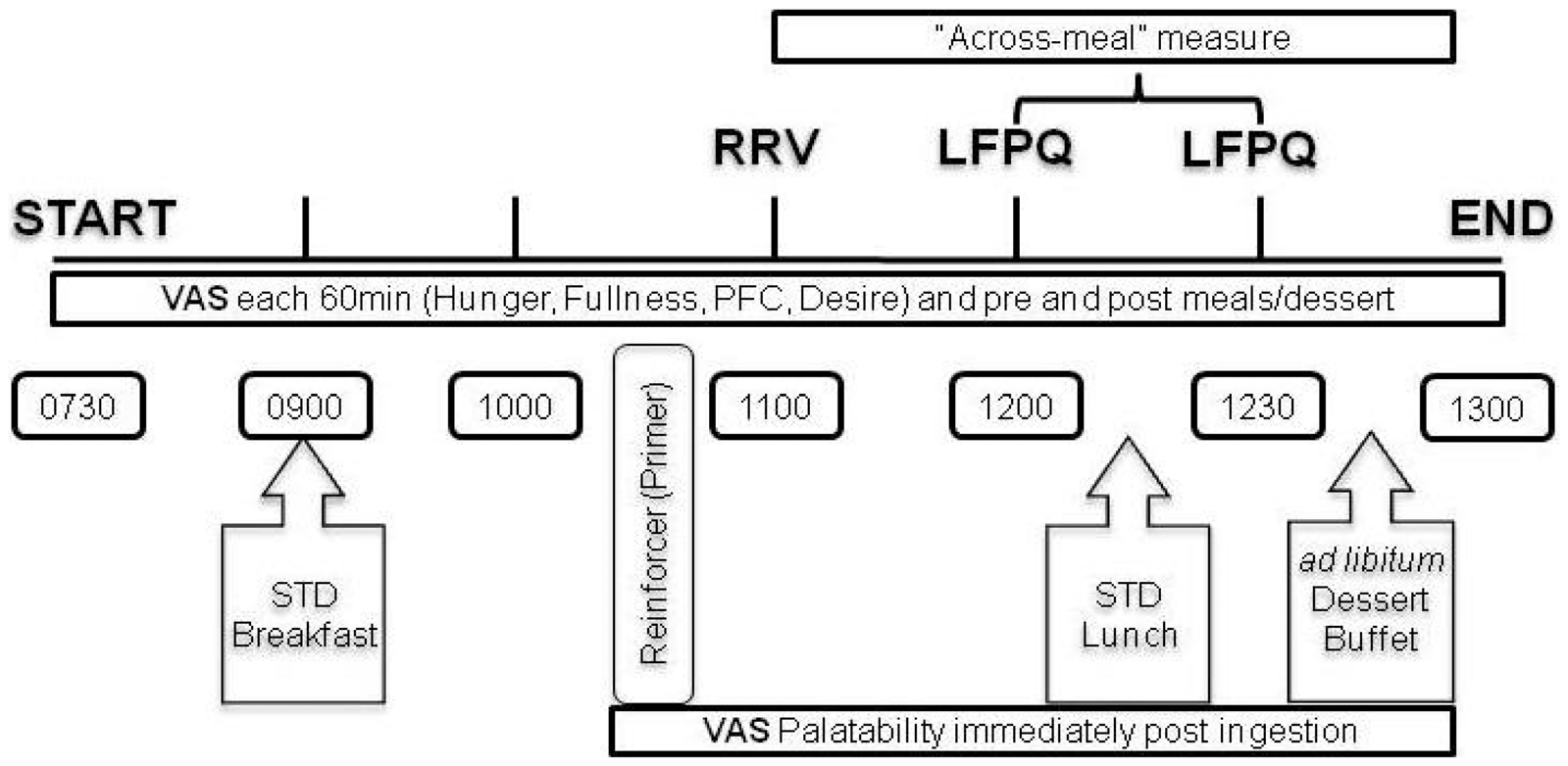
Protocol for the two testing sessions. Note that in order to perform the 24; the fasting period was slightly less than 24 hours due to the standardized consumption (100 kcal: 50 kcal snack and 50 kcal fruit) of the primers used in the RRV task.

Subjects arrived at 0800 fasted from 1900 the prior evening for the FED session and body weight and height were recorded. Subjects were then presented with a calorically-clamped breakfast which had to be consumed in 15 minutes at approximately 0900. Appetite measures (VAS) were taken first while fasted, and then every hour after completing the breakfast meal. Similarly, food ‘liking’ was measured immediately after the standardized breakfast, after the RRV primers, after lunch, and after the *ad libitum* dessert. The RRV computer task was administered at 1100 after consuming a 50 kcal primer each of the personalized snack and of the personalized fruit (see **Figure 1**). The second computer task—the LFPQ—measured explicit ‘liking’ and ‘wanting’ for food and was employed once at ∼1145, prior to eating lunch, and then once again immediately following lunch. Because there was a lunch meal in between the two measures of LFPQ, the term “across-meal” measure was used to describe this comparison of ‘wanting’ and ‘liking’. A standardized (calorically-clamped) lunch was served at ∼1200. At approximately 1230, after consuming lunch and after performing the post lunch LFPQ computer task, subjects were offered an *ad libitum* dessert buffet with a 30-minute time limit to eat as much or as little as desired. The session ended immediately after completing the buffet and VAS measures.

### Experimental manipulation of FASTED state

Subjects arrived at 0800 fasted from 1200 the prior day for the FASTED session body weight and height were recorded. Subjects were only allowed water during the 24 hours that defined the FASTED interval, which started outside of the lab at 1200 the day prior to testing lasting until 1100 on the day of testing in the lab. A self-reported checklist was used to verify compliance to the fast and indirect calorimetry was used as a complement to corroborate that the respiratory quotient was indicative of a fasted state (*i.e*., RQ<0.84). Appetite measures (VAS) were taken every hour. Food ‘liking’ was measured immediately after the food reinforcers for the RRV task (∼1100), and immediately following the lunch (∼1200) and *ad libitum* dessert (∼1230). The experimental design was a repeated measures and the only difference between the FASTED and FED sessions in the lab was that during the FASTED session subjects did not consume a breakfast on the day of testing.

### Measurements

#### Anthropometric measures

Height (HR-100 Height Rod; Tanita Corporation of America Inc. Arlington Heights, IL) and body weight (HR-100; BWB-800AS, Tanita Corporation, Arlington Heights, IL., USA) were measured after a 12 hour overnight fast, after voiding, while wearing a standard hospital gown.

#### Questionnaires: TFEQ

The TFEQ was administered during the initial screening process to determine the subject's individual attitude towards eating at a specific moment in time. It is understood that this questionnaire measures “trait” characteristics which are relatively stable, but are subject to change under periods of weight-loss, for example [40]. This 51 item questionnaire is an instrument that assesses three important attitudes to food: 1) chronic dietary restraint, which describes strategic dieting behavior, attitude to self-regulation, *etc*.; 2) disinhibition, which describes the vulnerability to lose control and over-consume and the responsiveness to the sight and smell of food; and 3) susceptibility to hunger, which describes internal and external loci of hunger [41].

#### Appetite and hedonic ‘liking’: visual analogue scales (VAS)

Appetite ratings were measured using a pen and paper on a 150-mm visual analogue scale (VAS) adapted from Hill and Blundell [42]. Desire to eat, hunger, fullness and prospective food consumption (PFC) were rated using the following questions: 1) “How strong is your desire to eat?” (Very weak- Very strong); 2) “How hungry do you feel?” (Not hungry at all- As hungry as I have ever felt); 3) “How full do you feel?” (Not full at all- Very full), and 4) “How much food do you think you could eat?” (Nothing at all- A large amount). Hedonic measures of ‘liking’ were similarly measured immediately following the ingestion of the personalized snack and fruit reinforcers (∼1045), and also immediately following each slice of pizza (∼1200), and finally after the *ad libitum* dessert (∼1300) with the following question: “How palatable was the meal?” (Not at all-Extremely). The trapezoid method was employed to measure the area under the curve (AUC) for all appetite-related variables, as previously described [43].

#### Standardized meals and personalized buffet

During the FED condition subjects were presented with a standardized breakfast, which had to be consumed within 15 minutes. The meal consisted of 2 pieces of whole wheat toast (*D'Italiano*®, 147 kcal), 17 grams of peanut butter (*Kraft* Smooth Peanut Butter®, 101 kcal), 15 grams of raspberry jam (*Smuckers*®, 50 kcal), and 250 ml of water for a total of 298 kcal. At 1200 in the FED and FASTED sessions, subjects were presented with a standardized lunch, which had to be consumed in 30 min. The meal consisted of 2 slices of cheese pizza (*Michelina's* Zap ‘Ems Gourmet®, 781 kcal). The *ad libitum* buffet consisted of 5 snack food items and 4 fruit/or vegetable items that were individualized—that is, the buffet items were previously indicated as being favorite food items on the food questionnaire from the RRV task. All items were presented on white foam plates and in standardized quantities (*e.g.* 70 g potato chip serving, 100 g apple serving, 100 g candy bar serving, *etc*.). Subjects were allotted 30 minutes to eat as much or as little of the buffet as they wanted and were told they could request more servings of any item. All food items were weighed to the nearest 0.1 gram (Scout Pro SP2001; Ohaus Corporation) and the difference calculated and subsequently analyzed with Food Processor SQL software (version 9.6.2.; ESHA Research).

#### Food reward: RRV and LFPQ computer tasks

The reinforcing value of a stimulus can be objectively observed as the increased (or decreased) willingness—a quantitative measure—to work (button presses) at obtaining food points via a progressive ratio computer task to obtain a desirable food stimulus (vs. some alternative). Specifically, for this study, computer software was used to set up a split screen that alternated between two different choices (a healthy food and palatable snack food) and was navigated with a mouse/pad (see [26]). Food points were earned by selectively working for the food item of choice. To obtain one fruit/vegetable point the reinforcement schedule was set at a progressive linear ratio with response requirements across five trials as follows: 4, 6, 8, 10, and 12. Thus, for example, at VR8 (3rd of 5 trials) the subject performed 8 button presses. For the snack foods the schedule was also set at a progressive ratio that doubled at each session as follows: 4, 8, 16, 32, and 64. The schedules of reinforcement were set to change differently between the two food stimuli in an effort to covertly disguise the discrepancy between the higher responding for the snack food. Note that 1 point was equivalent to 1 gram of the snack or fruit. Prior to performing the RRV task subjects were required to ingest a “primer” of the foods they picked as their favorite—approximately a 50 kcal portion of the snack and fruit reinforcers, respectively. Each subject was then shown what each food portion would look like if they worked entirely for the snack food or entirely for the fruit; this was done to help control for reward expectation [44] and previous research has shown that a primer can increase the ability to show differences in food reinforcement [45].

The LFPQ is designed to allow the separate and concurrent assessments of explicit ‘liking’ and ‘wanting’ for the same array of foods. This stimuli used in the task were photographic images of food varying in fat content (high or low) and taste (savory or sweet). These dimensions can be separated into four categories: high fat savory (HFSA), low fat savory (LFSA), high fat sweet (HFSW), and low fat sweet (LFSW). Five food items for a total of twenty different food stimuli represent each of the four categories. For the explicit measures of ‘liking’ and ‘wanting’, the 20 foods were rated according to “How pleasant would you find the taste of this food right now?“ and “How much do want some of this food right now?”.

#### Alliesthesia: ‘liking’ evaluation of food and food cues (images)

The phenomenon of alimentary alliesthesia was defined by Cabanac [46] as a change in the hedonic ‘liking’ of a stimulus that is modulated by the current bodily state—influenced by interoceptive signals and energetic needs. Briefly, alliesthesia was measured by Cabanac by the change in subjective pleasantness ratings for multiple ingestions of sweet tasting 25% solutions of aqueous glucose [13]. It is in this light that we measure the phenomenon of alliesthesia in the current study by evaluating the post-ingestive VAS ‘liking’ response to preferred food items (e.g. primers from the RRV task, pizza from the standardized lunch, and items from the dessert buffet) while under FED and FASTED conditions. Similarly, alliesthesia to food cues was measured by analogue scales in the LFPQ task for ‘liking’ of individual food images.

### Statistical methods

To test for differences in anthropometric variables, appetite, *ad libitum* EI, ‘liking’, RRV, and LFPQ across FED and FASTED sessions, repeated measures Analysis of Variance (ANOVAs) were used where Condition (FED vs FASTED) represented the within-subjects effects and sex represented the between subjects effects. Bivariate correlations were used to determine the strength of the relationship between the changes in food reward and the change in EI, and the relationship between TFEQ and food reward and EI; partial correlations controlling for age and sex were then used to follow up significant relationships. Statistical analyses were performed using SPSS version 17 (Chicago, SPSS Inc.). Results are presented as means ± SD and effects were considered significant at p<0.05.

## Results

As expected there were no significant changes in body weight or BMI between FED and FASTED conditions (see **Table 1**). Mean scores (maximum and minimum in parentheses) for Three Factor Eating Questionnaire (TFEQ) scores were 9 (±3.9) (4–17), 7.5 (±3.6) (2–13), and 7.6 (±3.0 (3–12), for dietary restraint, dietary disinhibition, and susceptibility to hunger, respectively.

There were significant differences (Condition effects) under the FASTED condition for all four measures of AUC (mm) for appetite (see **Table 1**). Relative to FED, *ad libitum* EI and all hedonic measures of ‘liking’ significantly increased under the FASTED condition. There were no significant sex or Condition x sex effects for variables of appetite or food hedonics (see **Table 1**).

As indicated by higher mean snack points (p<0.001) and higher mean snack responses (p<0.05) for the RRV task, significant Condition effects were noted for the RRV measure of food reward where preferred snack food was more reinforcing under the FASTED condition compared to FED (see **Table 1**). Relatedly, mean fruit points (p<0.05) and mean fruit responses (p<0.01) significantly decreased from FED to FASTED. There were no significant sex or time x sex effects for any RRV measure.

**Figure 2:**
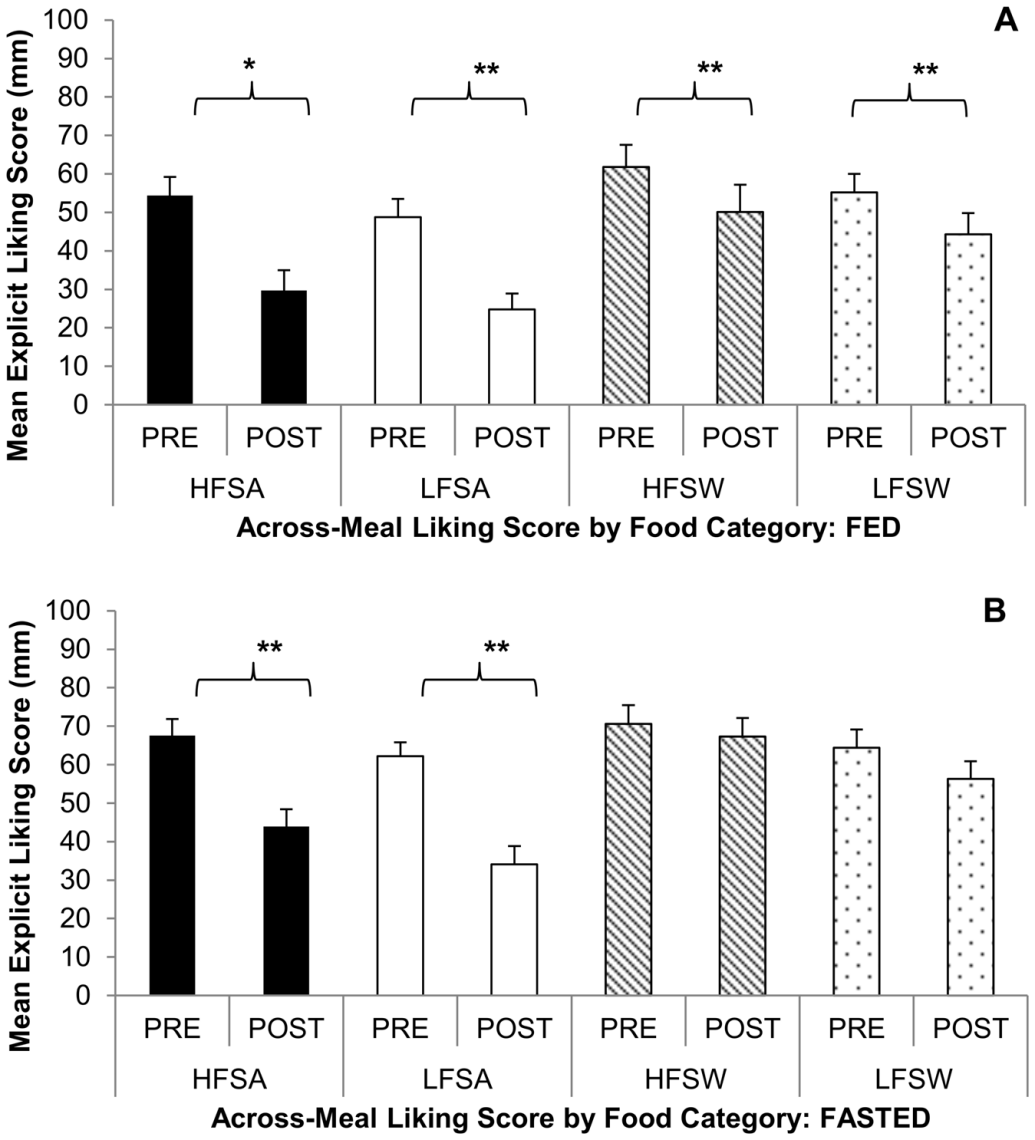
Results from the Leeds Food Preference Questionnaire for ‘liking’ of food images under FASTED and FED conditions. Showing the change in across-meal mean ‘liking’ score (± SD) for each of the four food categories: high fat savory (HFSA), low fat savory (LFSA), high fat sweet (HFSW) and low fat sweet (LFSW). In the FED condition (panel A) across-meal ‘liking’ for all four categories decreased. Panel B demonstrates the contrast of hedonic ‘liking’ ratings in the experimental condition and suggests the absence of negative alliesthesia to food images of the sweet food category after completing a 24 hour fast. *p<0.05, **p<0.001.

There were significant Condition effects as indicated by *pre-meal* increases in the LFPQ explicit ‘liking’ scores from FED to FASTED in the following: HFSA (54.4±18.6 vs. 67.5±16.4, p<0.05) and LFSW (55.2±17.3 vs. 64.4±18.2, p<0.05). Similarly, there were significant Condition effects for *post-meal* scores as indicated by increases in the explicit ‘liking’ scores from FED to FASTED for the following: HFSA (29.7±20.6 vs. 43.9±17.6, p<0.05), HFSW (50.6±27.6 vs. 67.3±18.8, p<0.01), and LFSW (44.3±21.4 vs. 56.3±17.9, p<0.05). There were significant Condition effects as indicated by *pre-meal* increases in all the LFPQ explicit ‘wanting’ scores for each specific food category from FED to FASTED: HFSA (49.8±18.8 vs. 68.9±18.2, p<0.05), LFSA (45.2±19.1 vs. 64.2±13.9, p<0.001), HFSW (50.3±22.2 vs. 69.1±20.4, p<0.005), and LFSW (47.6±15.1 vs. 59.9±17.9, p<0.05). Similarly, significant Condition effects were noted for the *post-meal* explicit ‘wanting’ rating from FED to FASTED, showing increases for each category: HFSA (21.8±20.5 vs. 40.2±22.1, p<0.01), LFSA (18.7±16.9 vs. 31.6±20.3, p<0.05), HFSW (38.3±23.4 vs. 62.8±22.0, p<0.005), and LFSW (34.7±18.9 vs. 52.9±18.9, p<0.01). In the FED condition there were significant *across-meal* decreases in explicit ‘liking’ for all four food categories: HFSA (p<0.05), LFSA (p<0.001), HFSW (p<0.001), and LFSW (p<0.001) (see **Figure 2**). In the FASTED condition, there were only *across-meal* decreases in explicit ‘liking’ for HFSA (p<0.001) and LFSA (p<0.001), while no significant decreases were noted for the two sweet categories (see **Figure 2**). The only noted sex effect for the LFPQ variables was for post-meal ‘wanting’ of HFSA, where men wanted HFSA foods more than women post-meal; there were no time x sex interactions for any LFPQ variables.

Bivariate correlations revealed that the noted increase in *ad libitum* EI under the FASTED condition was positively correlated with the change in the AUC appetite score for desire to eat (r = 0.55, p = 0.04). Partial correlations controlling for sex revealed: that TFEQ disinhibition scores were positively correlated with snack points earned under the FED condition (r = 0.56, p<0.05); and that under the FASTED condition, disinhibition was positively correlated with snack points (r = 0.55, p<0.05) and snack responses (r = 0.52, p<0.05). There were no other significant correlations between variables.

## Discussion

This study demonstrated evidence of acute changes in the rewarding qualities of preferred foods after a 24-hour fast. *Ad libitum* EI from the buffet increased 74% from FED to FASTED and food was not only subjectively rated with higher post-ingestive ‘liking’ scores (VAS) in the FASTED condition, but food *images* were also perceived as more liked both pre and post lunch, suggestive of alliesthesia for orosensory and visual stimuli. Across-meal measures of the LFPQ for ‘liking’ in the FASTED condition demonstrated an attenuated score for savory foods, but sweet foods maintained a strong hedonic saliency by failing to show the decrease in ‘liking’ scores noted in the FED condition from pre- to post-meal.

Intuitively, one would anticipate that energy deprivation results in increased hunger ratings. But evidence suggests that as the fasting period is carried over from hours to days the appetite response can normalize to baseline levels previously noted in the fed state after approximately 4-5 days of fasting [5], [47]. Similar seemingly discrepant results have been found with less dramatic methods of energy deprivation, namely very low calorie diets, where on average subjects reported decreased appetite during the intervention—described as “less food less hunger” [48]–[50]. In a study with a fasting period (19 hours) comparable to our design, subjects without clinical eating pathology did not eat significantly more when allowed access to *ad libitum* feeding [2]. Compared to FED, the appetite response to the current 24-hour fast was significantly impacted for all four AUC appetite measures. Most affected was hunger, which increased by 69%, followed by 50% and 30% increases in prospective food consumption and desire to eat, respectively, along with a 47% decrease in fullness. After 24 hours of fasting we demonstrate a significant 74% increase in mean *ad libitum* EI (kcal), which was positively correlated with increased AUC desire to eat scores. A similar study found significantly increased appetite scores following a 36-hour fast but they noted that when *ad libitum* feeding was reinstated there was only a 20% increase in EI, and this was unrelated to the changes in appetite [4].

What is interesting is that our data is suggestive of alliesthesia for preferred food stimuli; that is, subjects rated ‘liking’ of the *ad libitum* dessert significantly higher under the FASTED condition even after eating 74% more energy (see **Table 1**). Corroborating these results are findings from deprivation periods of much shorter duration. By manipulating the period of energy deprivation with two separate test days, one day with a 3.5 hour period of deprivation and another with an overnight fast of approximately 12–15 hours, Speigel et al. [51] found that the deprivation period enhanced palatability, again consistent with the concept of alliesthesia. A strength of our findings lies in the fact that although neither the macronutrient nor the item content of the dessert buffet were controlled between subjects, there were no significant differences in percent fat or carbohydrate intake between the *ad libitum* EI in the FED and FASTED conditions (see **Table 1**). This observation is somewhat analogous to controlling for macronutrient intake and allows for better comparisons in overall changes in ‘liking’, either measured by VAS or by the LFPQ, thereby permitting a better analogue when comparing our results with previous work on alliesthesia (*e.g.* [46], [52]). To be sure, our ‘liking’ measures obtained immediately post-consumption of the preferred food items do not mimic the pure measures of ‘liking’ obtained by Cabanac with a 25% aqueous solution of glucose [13], but our findings are indeed consistent with the concept of alliesthesia.

Another interesting question that arises is whether there are differences in food reward or underlying trait differences (*e.g.* TFEQ) that can be attributed to the energy deprivation or to the noted increase in EI and palatability. The RRV task employed in the current study demonstrated that relative to FED, there were FASTED increases in the reinforcing value of palatable food as evidenced by significantly higher points earned and responses made for the snack item versus the preferred fruit (see **Table** **1**). To our knowledge there is only one other study using the RRV task under comparable levels of energy deprivation; after ∼13 hours of fasting food also became more reinforcing for normal weight non-dietary restrained females [34]. What is interesting is that there is some evidence that this increase in the reinforcing value of food can predict *ad libitum* EI, independently of rated ‘liking’, or hedonic ratings of foods [53], [54]. Our data, however, did not demonstrate a relationship between RRV and EI. That is, although snack foods were significantly more reinforcing under the FASTED condition, we found no indication that this increased desire or motivation to obtain more energy dense foods was related to the increased *ad libitum* EI noted under the FASTED condition. Regarding eating behavior traits, we have replicated recent findings that TFEQ scores for dietary disinhibition are related to food reinforcement [26]. Specifically, our data show a positive association between disinhibition scores and the ‘wanting’ for palatable energy dense snack food in the RRV task. Although the relationship between RRV, disinhibition and reward responsiveness remains to be better defined, there seems to be more consistent data confirming a positive relationship between BMI, or weight gain, and disinhibition scores [55]. This would suggest that individuals who score high in disinhibited eating may be more vulnerable to increases in food reward and food palatability ratings following a period of food deprivation, which may put them at greater risk of subsequently overconsuming food under such circumstances.

The LFPQ is different from the RRV task in that it allows the separate and concurrent assessments of explicit ‘liking’ and ‘wanting’ for the same target stimuli [56]. In our sample, explicit ‘wanting’ and ‘liking’ of food images increased under the FASTED relative to FED condition: except for the lack of change in ‘liking’ for LFSW foods, both pre-meal scores and post-meal scores significantly increased for all four food categories. In previous work with the LFPQ measuring ‘wanting’ and ‘liking’, once in a hungry state (3–4 hours post-prandial) and then after eating a lunch meal on the same test day [57], it was shown that ‘liking’ for sweet foods did not decrease as much as that for fat foods. Here we show similar results in the FED condition, but under the FASTED condition only images of savory foods decreased in ‘liking’, while images of sweet foods maintained significantly heightened across-meal ‘liking’ (see **Figure 2**). Our results are in agreement with recent imaging studies showing a robust activation of brain reward areas to sucrose taste [58] and to images of high calorie foods [18], particularly after a period of fasting. Similar results were found in a study investigating alliesthesia to food images and found that foods rated with the highest ‘liking’ scores (*i.e.* desserts) were less affected by energy deprivation than the savory foods [22], suggesting that the hedonic value of a food stimulus may predict the magnitude of alliesthesia. Limited data on alliesthesia to food images exists: no effect of deprivation on ratings of ‘liking’ has been reported [59], while others have reported higher ‘liking’ ratings under hungry versus sated subjects [22], [23], [60], [61]. It should be noted that the explicit ‘liking’ responses from the LFPQ as they relate to alliesthesia are distinct from the ‘liking’ measures we obtained from the VAS palatability measure immediately post-ingestion. That is, the ‘liking’ of food images is a composite of learned hedonic features of the food (e.g. previous exposure of taste, texture, post-ingestive consequences, etc.), whereas, the VAS ‘liking’ is a momentary evaluation of sensory qualia of the food.

Limitations for the current study are the fact that the fasting period was out of the laboratory and therefore there was the possibility that contrary to their self-reported fast, some subjects did not precisely follow the protocol. The relatively small number of moderately overweight but otherwise healthy subjects limits the scope of the current findings to similar populations. Also, because pizza was served as the standardized lunch, there could have existed a sensory specific satiety to savory food cues, which may partially explain the decreased post-prandial ‘liking’ of savory food images. It is worthwhile to restate here that ‘wanting’ of savory foods after the lunch was higher in males, thus we cannot discount sex-related differences in food evaluation [62], [63]. Some of the strengths are in the randomized crossover design, that women were tested in the same phase of the menstrual cycle, and that the food items were personalized. Finally, to confirm our findings, more research is needed to clarify the role that sex has in responding to various degrees of negative energy balance, and in the subsequent response to food or food-related cues.

As a result of a 24-hour complete fast, preferred foods were both wanted and liked significantly more than in the fed state. Scores for appetite were dramatically affected, pushing up hunger, desire to eat and PFC, while simultaneously attenuating fullness. Higher disinhibition scores correlated with responding for palatable snack food stimuli in the RRV task, further indicating that RRV has strong ties with impulsivity and food sensitivity. Considering that there was a 74% increase in EI under the FASTED condition, our data suggest that alliesthesia was demonstrated by heightened hedonic ratings of ‘liking’ following this much larger eating episode compared to the FED condition. The results from the LFPQ were in agreement with the abovementioned finding, whereby there was an increase in explicit ‘wanting’ and ‘liking’ to food images after 24 hours of fasting. The current study is also timely due to the recent, but still nascent findings, that alternating days of 24 hours of fasting may confer protection against coronary artery disease [64] and improve insulin sensitivity [65] in humans. More research is needed to better describe the degree that these measures of food reward are conceptually unique or structurally overlapping; furthermore, given the surge in brain imaging research and recent controversy regarding food addiction [66], [67], it becomes clear that validated computer tasks like those discussed here will compliment a more sophisticated biological and behavioral understanding of the human response to energy deprivation.
